# Supplementary material for: The mechanism for the enhanced piezoelectricity in multi-elements doped (K,Na)NbO3 ceramics
Source: Nat Commun. 2021 Feb 9;12:881. doi: 10.1038/s41467-021-21202-7 (PMC7873261; doi:10.1038/s41467-021-21202-7)
Supplement: Supplementary file 1 — Supplementary Information [file 41467_2021_21202_MOESM1_ESM.pdf]

The mechanism for the enhanced piezoelectricity in multi-elements doped (K,Na)NbO_3 ceramics

Xiaoyi Gao, Zhenxiang Cheng, Zibin Chen, Yao Liu, Xiangyu Meng, Xu Zhang, Jianli Wang, Qinghu Guo, Bei Li, Huajun Sun, Qinfen Gu, Hua Hao, Qiang Shen*, Jinsong Wu*, Xiaozhou Liao, Simon P Ringer, Hanxing Liu, Lianmeng Zhang, Wen Chen, Fei Li*, Shujun Zhang*

Correspondence to: Q.S. (email: sqqf@whut.edu.cn), J.S.W. (email: wujs@whut.edu.cn), F.L. (email: ful5@xjtu.edu.cn), and S.Z. (email: shujun@uow.edu.au)

Supplementary Figure 1. The FE-SEM images of multi-elements and single-element doped KNN ceramics.

Supplementary Figure 2. The relationship between intrinsic and extrinsic contributions to piezoelectricity.

Supplementary Figure 3. The synchrotron X-ray diffraction patterns and refinement results for KNN-Sb ceramic over a temperature range of 123–373 K.

Supplementary Figure 4. The synchrotron X-ray diffraction results for KNN-Sb ceramic over temperature range of 123–373 K.

Supplementary Figure 5. The synchrotron X-ray diffraction patterns and refinement results for KNN-Bi,Sb,Zr ceramic over a temperature range of 123–423 K.

Supplementary Figure 6. The synchrotron X-ray diffraction results for KNN-Bi,Sb,Zr ceramic over temperature range of 123–423 K.

Supplementary Figure 7. The contributions of the A-site and oxygen vacancies to the microstructure based on the first principles calculations.

Supplementary Figure 8. The temperature dependence of relative dielectric permittivity and loss factor for the studied doped KNN ceramics.

Supplementary Figure 9. The dielectric behavior of multi-elements and single element doped KNN ceramics over cryogenic temperature range of 120–300 K.

Supplementary Figure 10. The X-ray diffraction results for KNN-Bi,Sb,Zr ceramic over temperature range of 500–800 K.

Supplementary Figure 11. The dielectric loss factor and relative dielectric permittivity as a function of frequency for KNN-Bi,Sb,Zr ceramic over a temperature range of 153–293 K.

Supplementary Figure 12. The phase field simulations of the microstructure evolution as a function of applied electric field along the [100] direction for pure tetragonal phase structure.

Supplementary Figure 13. The measurement results of piezoelectric strains for KNN-Bi,Sb,Zr and KNN-Sb ceramics at 4kV/cm.

Supplementary Figure 14. The domain structure images of KNN-Sb and KNN-Bi,Sb,Zr ceramics.

Supplementary Figure 15. The supercell of pure KNN that used to perform first-principles calculations.

Supplementary Table 1. Some major properties of multi-elements and single element doped KNN ceramics.

Supplementary Figures

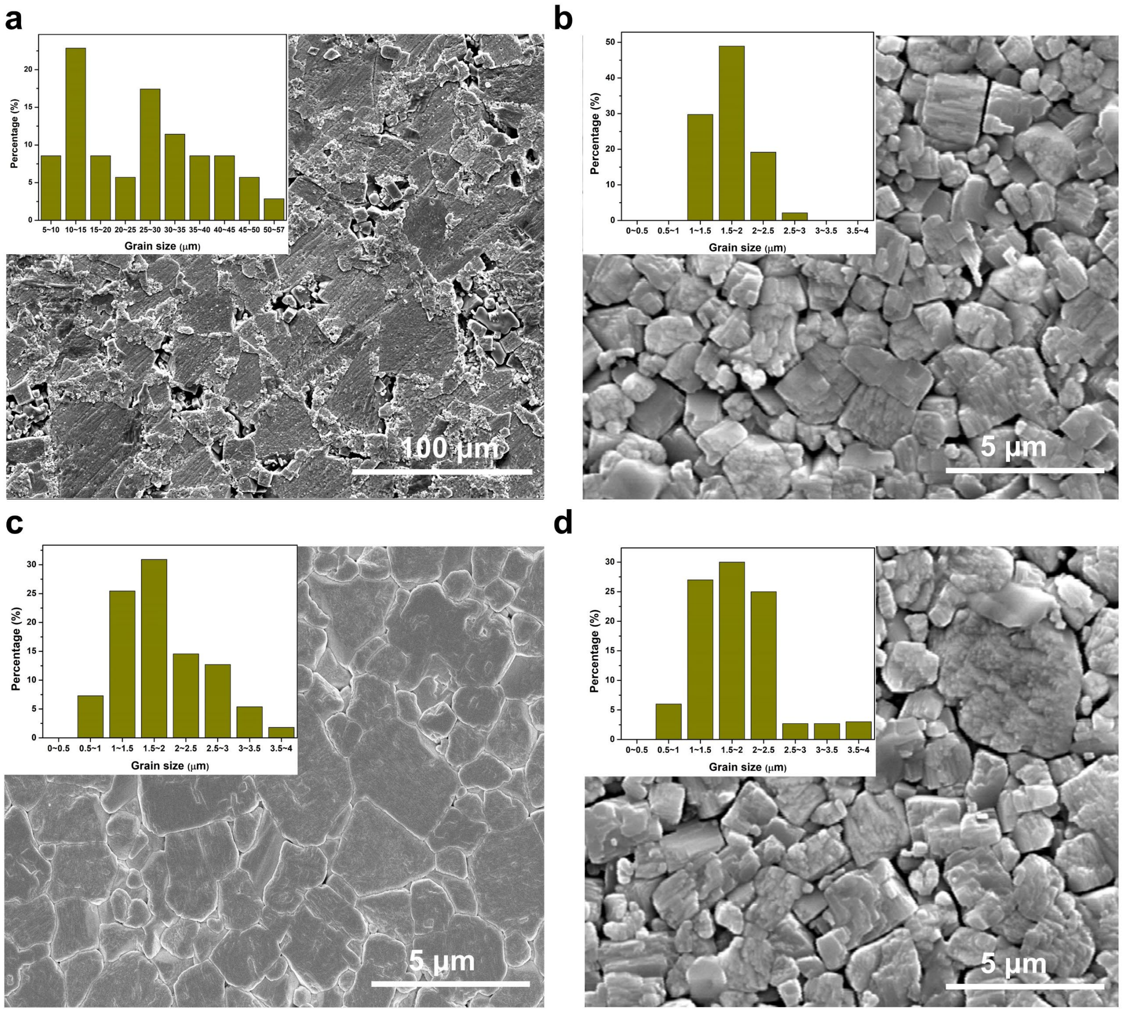

Supplementary Figure 1. The FE-SEM images of multi-elements and single-element doped KNN ceramics. (**a**) The FE-SEM image for multi-elements doped KNN ceramic (KNN-Bi,Sb,Zr). (**b-d**) The FE-SEM images for KNN-Bi, KNN-Sb, KNN-Zr ceramics, respectively.

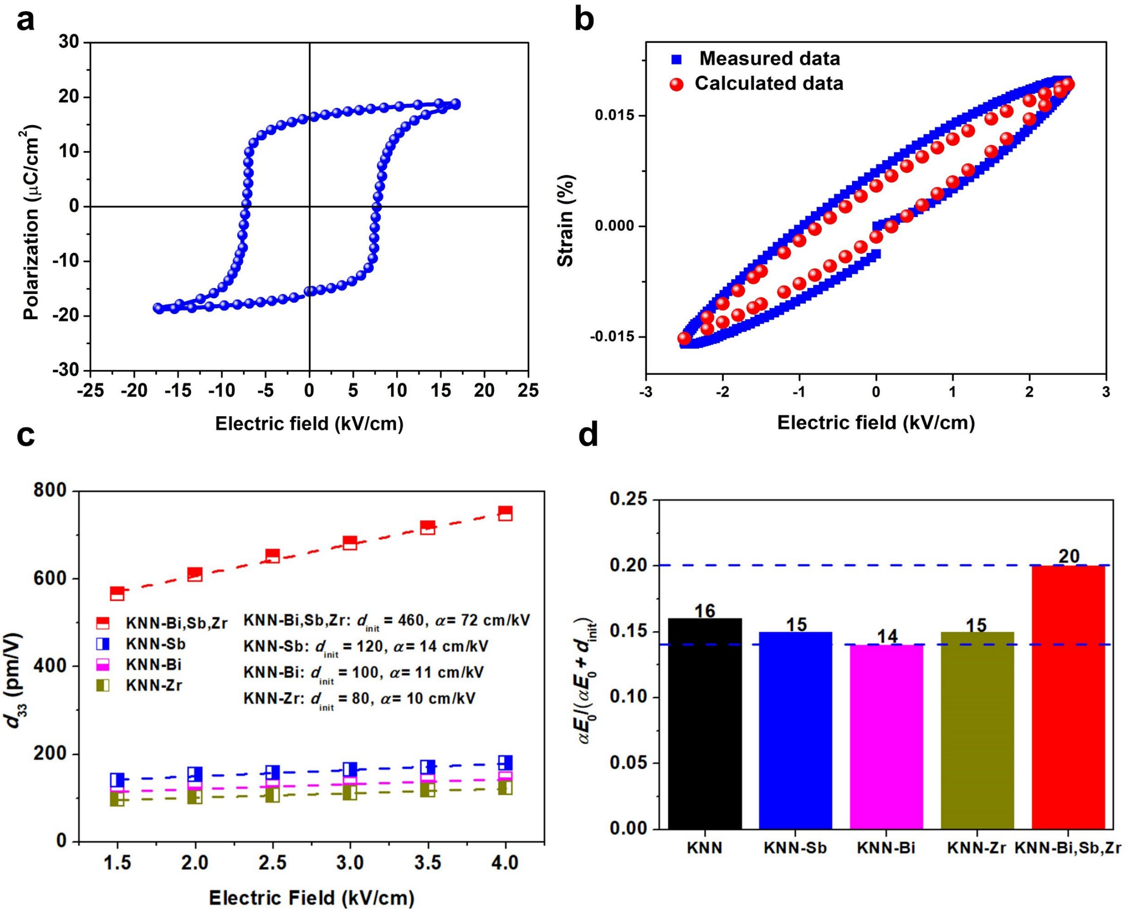

Supplementary Figure 2. Rayleigh study and the intrinsic/extrinsic contributions to the piezoelectricity. (**a**) The ferroelectric hysteresis loop of KNN-Bi,Sb,Zr ceramic. (**b**) Comparison between the measured and calculated strain-versus-electric field hysteresis loop of KNN-Bi,Sb,Zr ceramic below the subcoercive field. (**c**) The ac electric field–dependent piezoelectric coefficient d_{33} and Rayleigh parameters for KNN-based ceramics. (**d**) The ratio of extrinsic contribution $\alpha E_0/(\alpha E_0+d_{\mathrm{init}})$ for KNN-based ceramics.

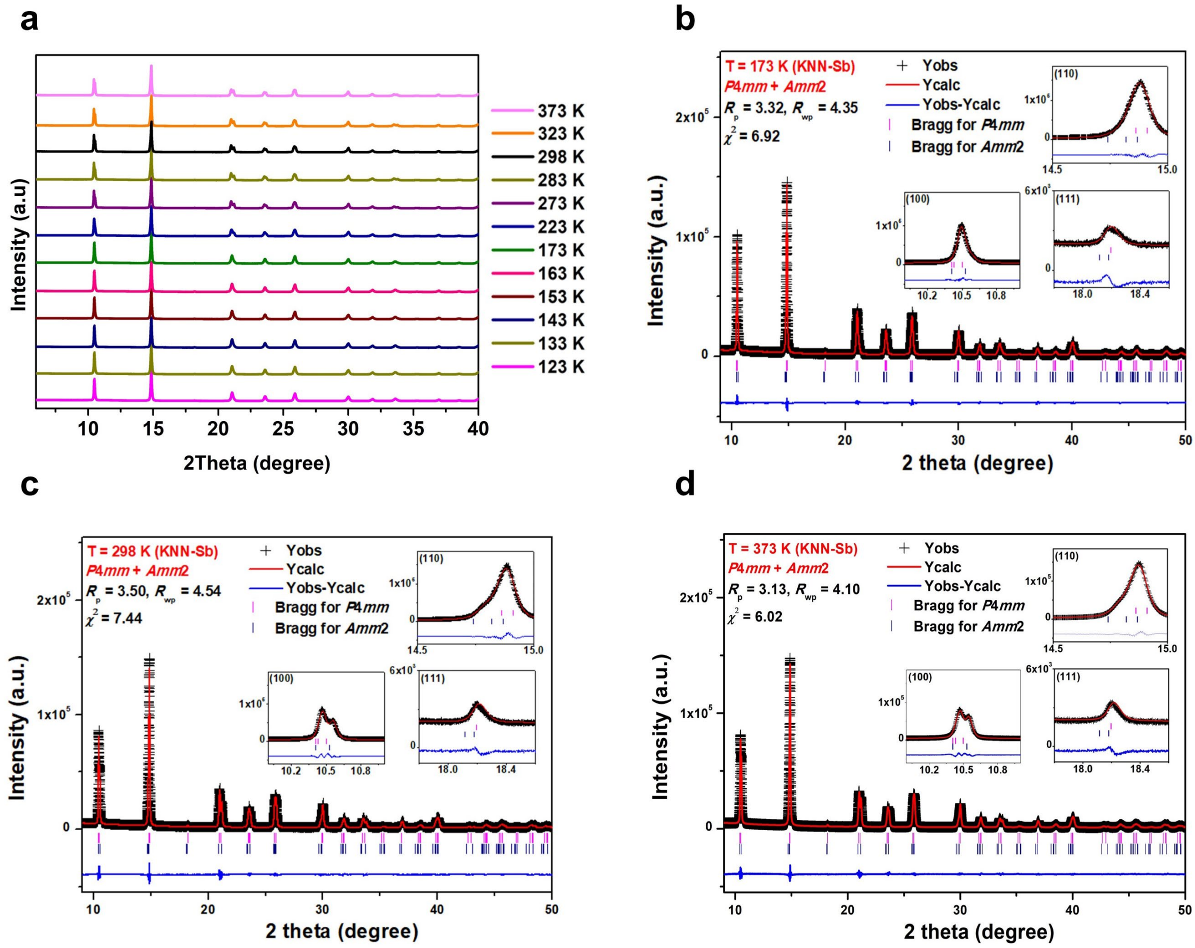

Supplementary Figure 3. The synchrotron X-ray diffraction patterns and refinement results for KNN-Sb ceramic over a temperature range of 123–373 K. (**a**) The synchrotron X-ray diffraction patterns. (**b-d**) The Rietveld refinements results for 173 K, 298 K, and 373 K, respectively.

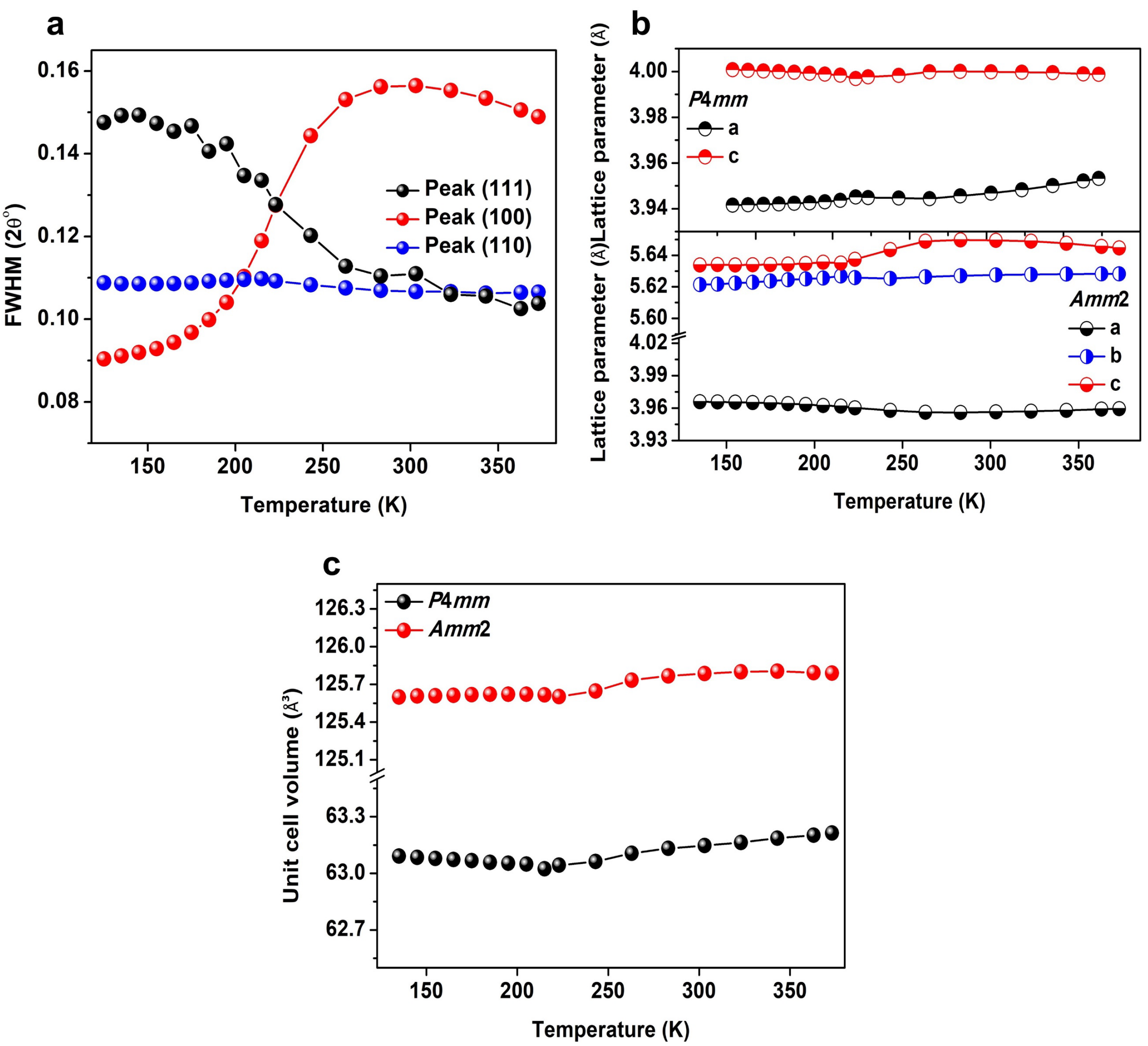

Supplementary Figure 4. The synchrotron X-ray diffraction results for KNN-Sb ceramic over temperature range of 123–373 K. (**a**) The full width at half maximum (FWHM) values of (111), (100), and (110) peaks. (**b**) The lattice parameters of the orthorhombic and tetragonal phases. (**c**) The unit cell volume of orthorhombic and tetragonal phases.

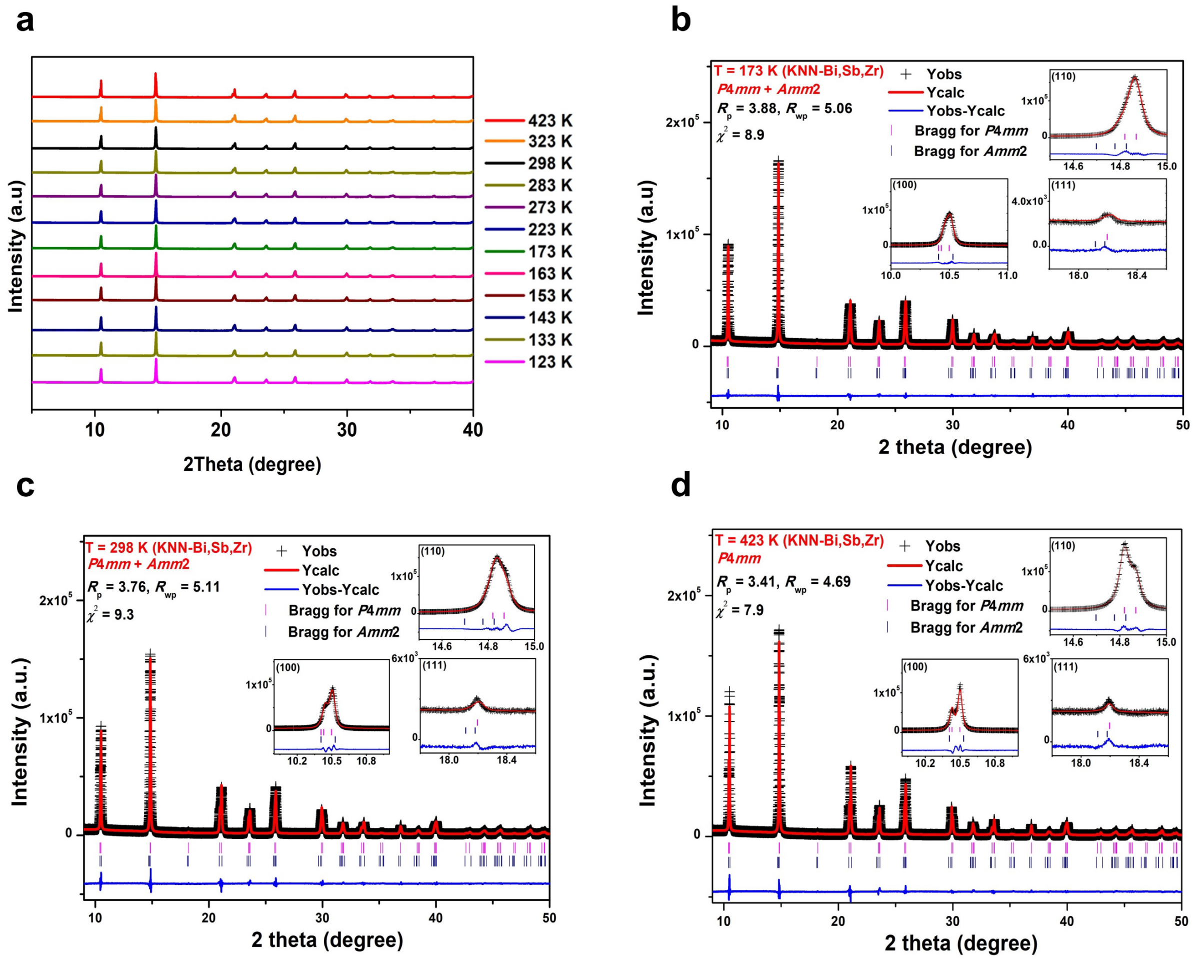

Supplementary Figure 5. The synchrotron X-ray diffraction patterns and refinement results or KNN-Bi,Sb,Zr ceramic over a temperature range of 123–423 K. (**a**) The synchrotron X-ray diffraction patterns. (**b-d**) The Rietveld refinements results for 173 K, 298 K, and 423 K, respectively.

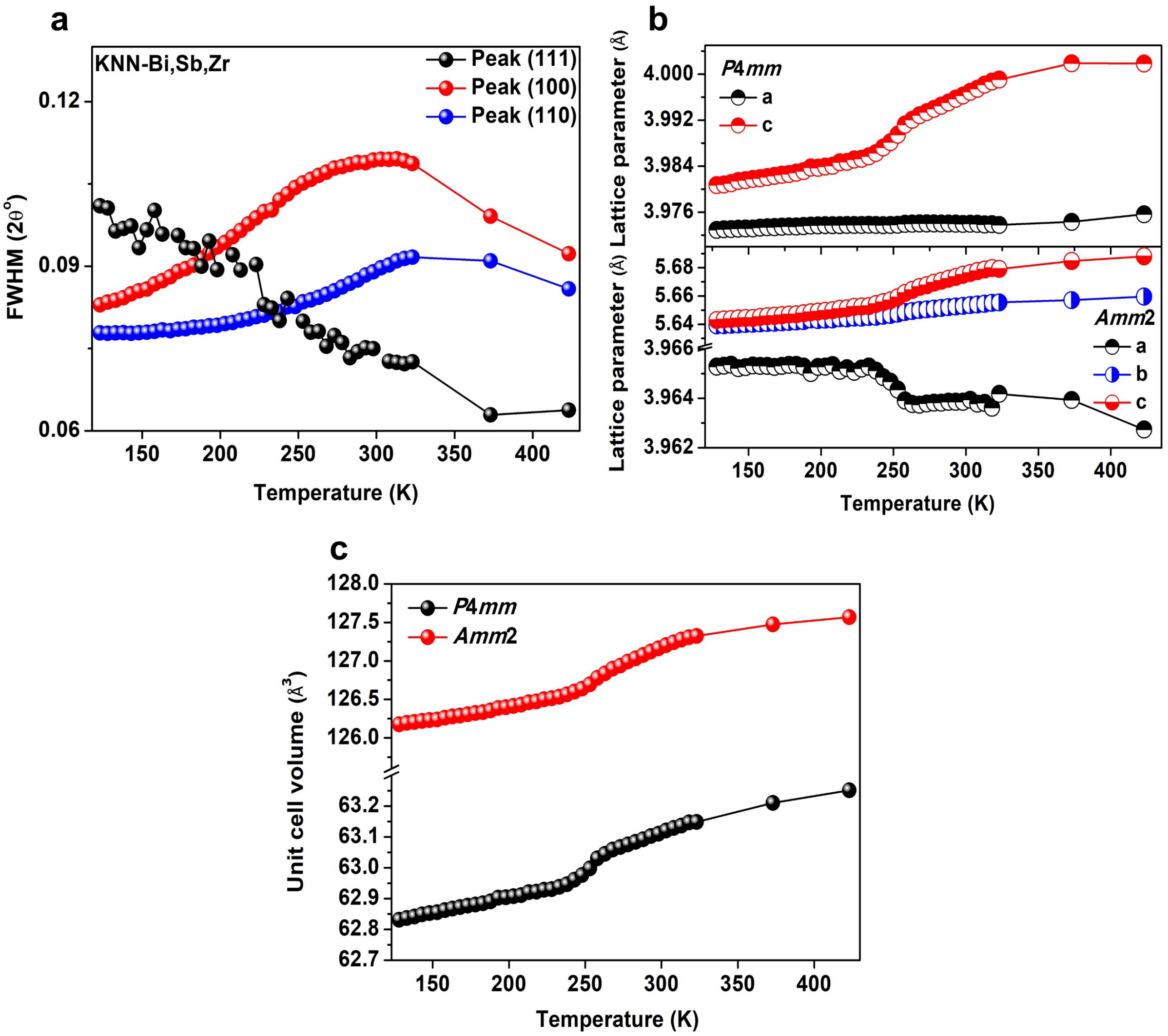

Supplementary Figure 6. The synchrotron X-ray diffraction results for KNN-Bi,Sb,Zr ceramic over temperature range of 123–423 K. (**a**) The full width at half maximum (FWHM) values of (111), (100), and (110) peaks. It is interesting to note that the FWHM values of KNN-Bi,Sb,Zr sample are lower than those of KNN-Sb counterpart (Supplementary Fig. 4), indicating the tetragonality or orthorhombicity of KNN-Bi,Sb,Zr sample is decreased comparing to the KNN-Sb sample. (**b**) The lattice parameters of the orthorhombic and tetragonal phases. (**c**) The unit cell volume of orthorhombic and tetragonal phases.

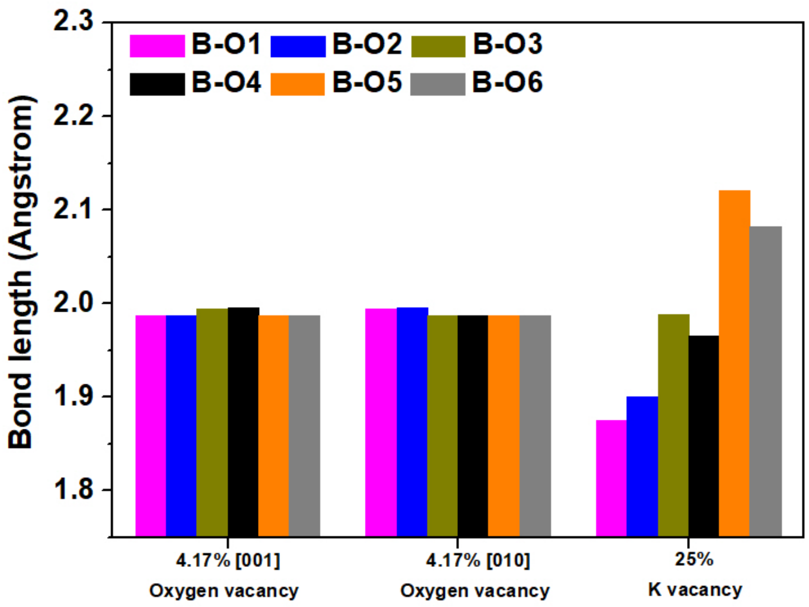

Supplementary Figure 7. The contributions of the A-site and oxygen vacancies to the microstructure based on the first principles calculations. The length of the six B-O bonds of KNNs approaches the same value after adding oxygen vacancies, while the difference in the length of the six B-O bonds is still very large by adding A-site vacancy, being similar to the pure KNN.

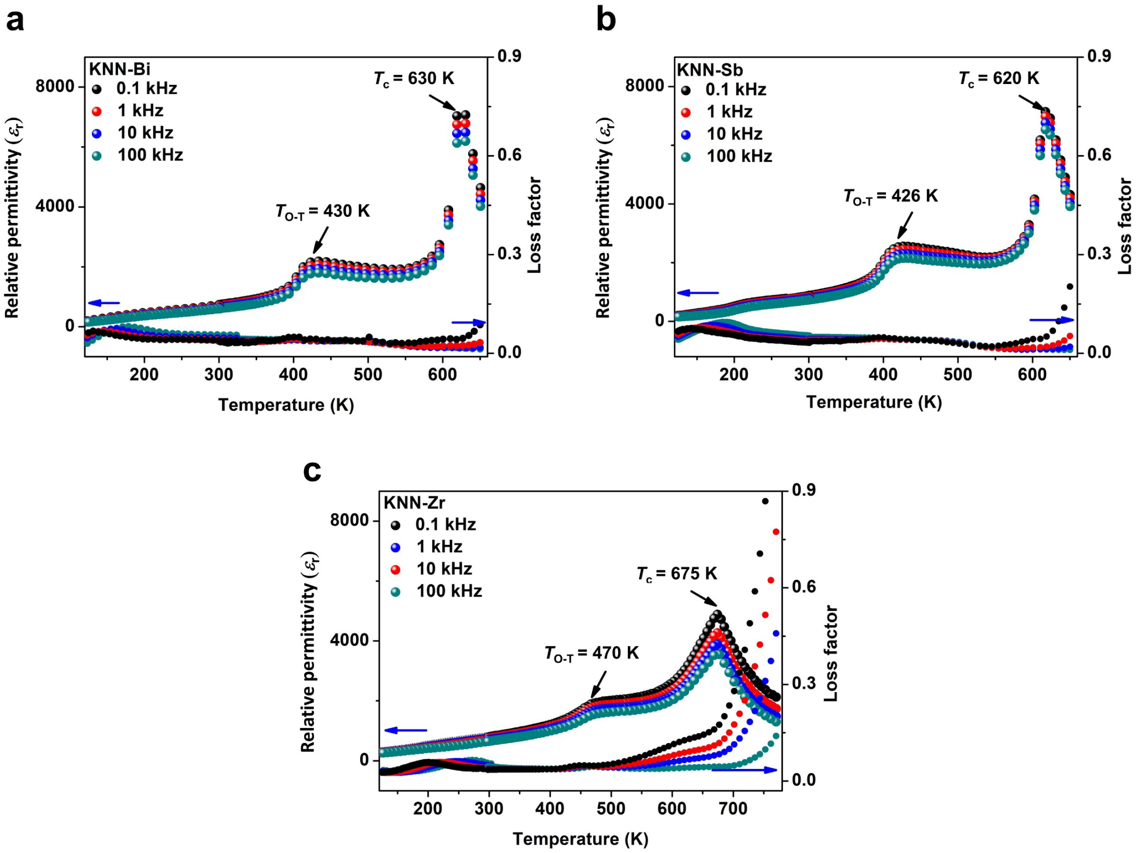

Supplementary Figure 8. The temperature dependence of relative dielectric permittivity and loss factor of the studied doped KNN ceramics. (**a**) KNN-Bi ceramic, (**b**) KNN-Sb ceramic, (**c**) KNN-Zr ceramic.

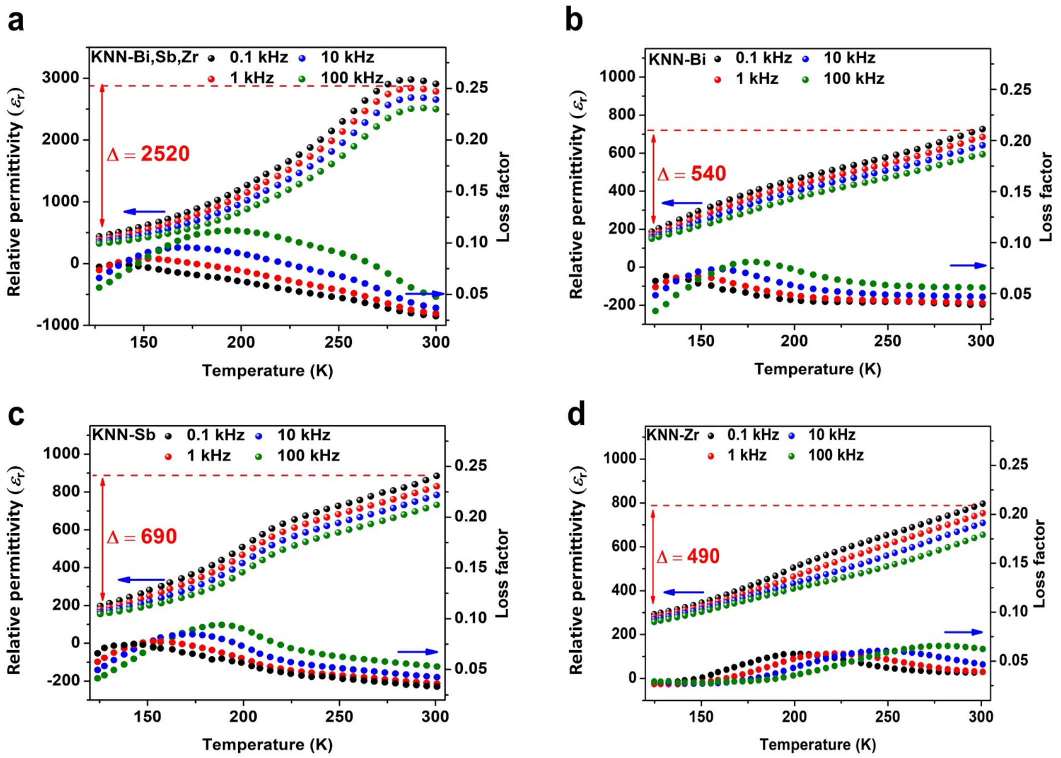

Supplementary Figure 9. The dielectric behavior of multi-elements and single-element doped KNN ceramics over cryogenic temperature range of 120–300 K. (**a**) KNN-Bi,Sb,Zr ceramic, (**b**) KNN-Bi, (**c**) KNN-Sb, (**d**) KNN-Zr ceramics. The temperatures of loss factor peaks in single element doped KNNs are overlapped over the temperature range of the board loss factor peak in multi-elements doped KNN ceramic (KNN-Bi,Sb,Zr).

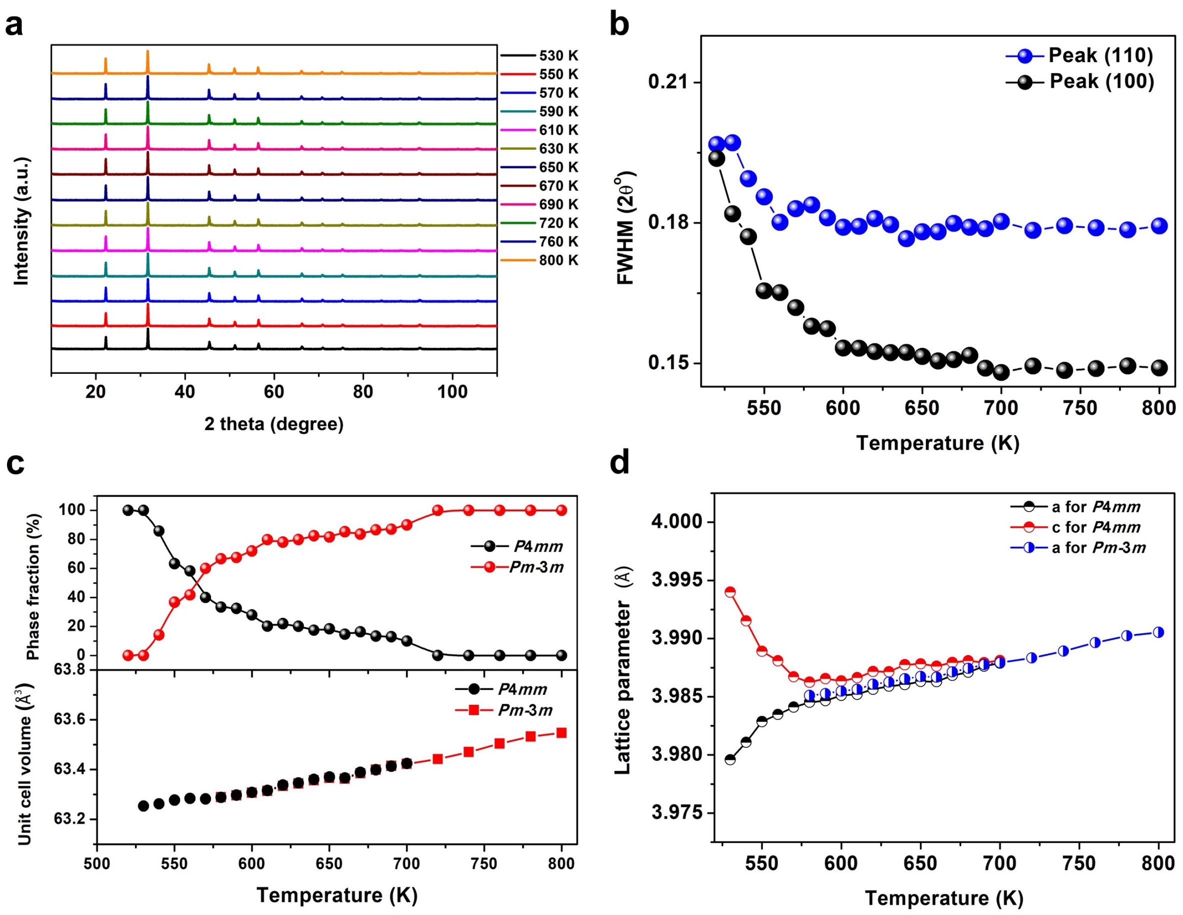

Supplementary Figure 10. The X-ray diffraction results for KNN-Bi,Sb,Zr ceramic over temperature range of 500–800 K. (**a**) The X-ray diffraction patterns for KNN-Bi,Sb,Zr ceramic. (**b**) The full width at half maximum (FWHM) values of (110), (100) peaks. (**c**) The phase fraction and unit cell volume of the tetragonal and cubic phases. The temperature of phase transition from tetragonal to Cubic phase is around 550 K, while the tetragonal phases yet exists over temperature above the T_c. (**d**) The lattice parameters of tetragonal and Cubic phases.

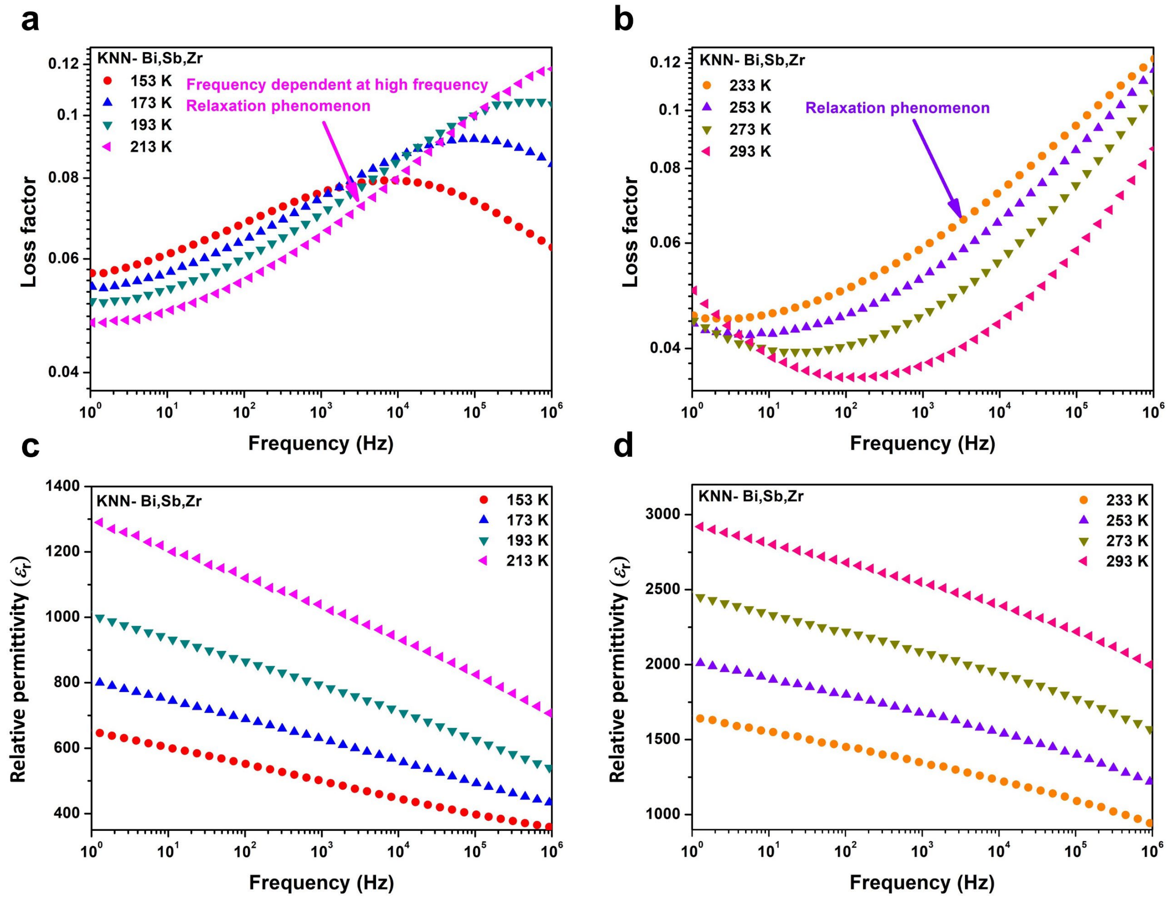

Supplementary Figure 11. The dielectric loss factor and relative dielectric permittivity as a function of frequency for KNN-Bi,Sb,Zr ceramic over temperature range of 153–293 K. (**a-b**) dielectric loss factor, (**c-d**) relative dielectric permittivity.

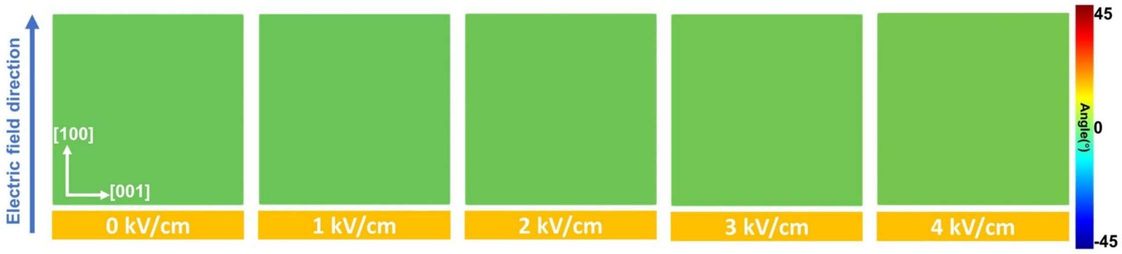

Supplementary Figure 12. The phase field simulation of the microstructure evolution as a function of applied electric field along the [100] direction for pure tetragonal phase structure. The colour bar shows the angle of polar vector direction off the [001] direction. The green colour shows the polar vectors are along the [001] spontaneous polarization direction of tetragonal phase structure (the angle is 0º). The subtle variation of the colour with increased external electric field indicates that the average polar vector variation is very small as function of electric field.

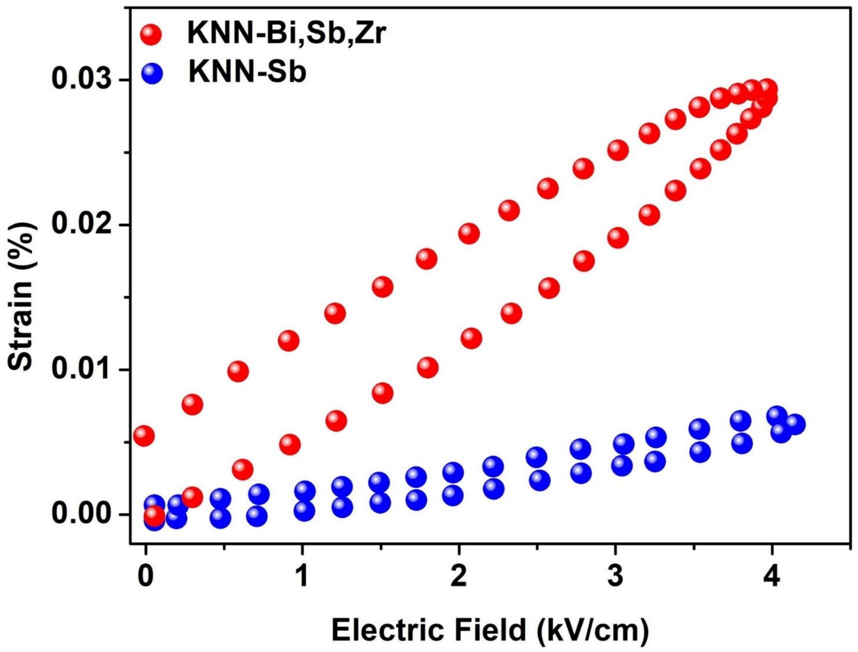

Supplementary Figure 13. The measurement results of piezoelectric strains for KNN-Bi,Sb,Zr and KNN-Sb ceramics at 4kV/cm.

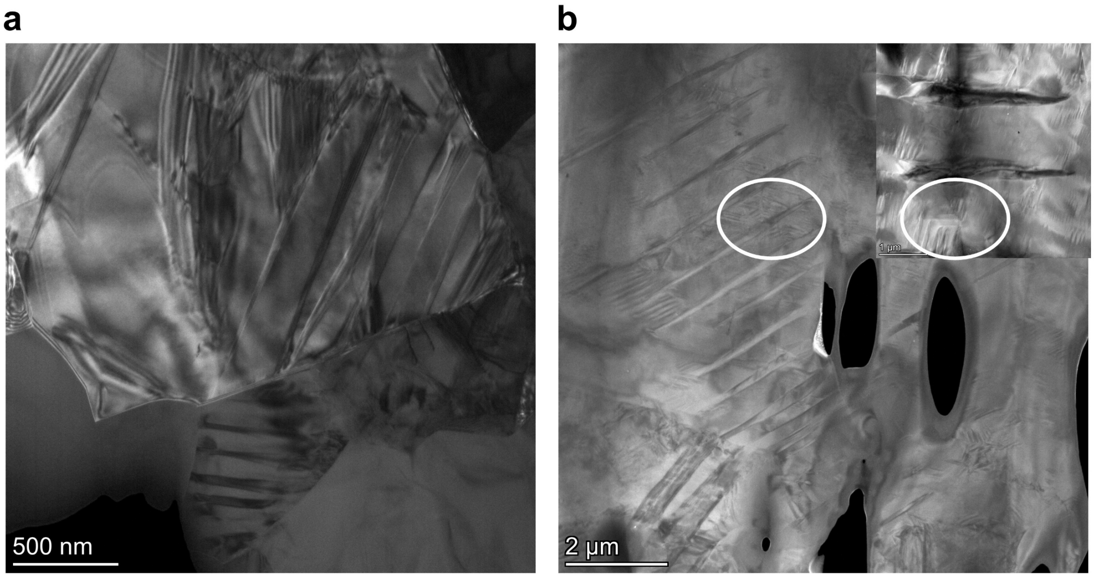

Supplementary Figure 14. The domain structure images of KNN-Sb and KNN-Bi,Sb,Zr ceramics. (**a**) The domain structure of KNN-Sb ceramic. The domain size is about 100 nm in the small grain. (**b**) The domain structure of KNN-Bi,Sb,Zr ceramic. The domain is about 500 nm in large grain, which couple with the hierarchical domain structure (mark with the white circle).

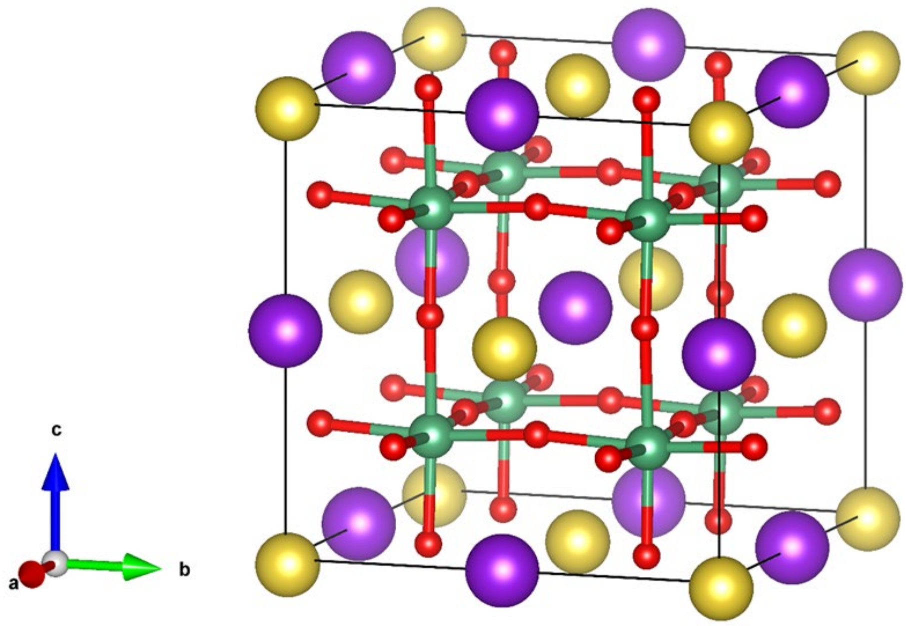

Supplementary Figure 15. The supercell of pure KNN that used to perform first-principles calculations. The purple, yellow, green, and red atoms are K, Na, Nb, and O atoms, respectively. The supercell with the uniform distribution of A-site cations along the a, b, and c directions was used in the calculations.

Supplementary Table

Supplementary Table 1. Some major properties of multi-elements and single-element doped KNN ceramics.

Material	ε_r (at 1kHz)	Loss factor (at 1kHz)	T_c (K)	d_{33} (pC/N)	Relative density (%)	Average grain size (μm)	E_c (kV/cm)
KNN-Bi,Sb,Zr	2900	0.021	544	520	>96	25	8
KNN-Bi	690	0.025	630	140	>94	2.0	18
KNN-Sb	810	0.022	620	160	>96	2.0	11
KNN-Zr	680	0.032	675	120	>94	2.0	13
